# Antitumor effects of low‐dose tipifarnib on the mTOR signaling pathway and reactive oxygen species production in HIF‐1α‐expressing gastric cancer cells

**DOI:** 10.1002/2211-5463.13154

**Published:** 2021-04-08

**Authors:** Noriyuki Egawa, Tomokazu Tanaka, Shohei Matsufuji, Kohei Yamada, Kotaro Ito, Hiroshi Kitagawa, Keiichiro Okuyama, Yoshihiko Kitajima, Hirokazu Noshiro

**Affiliations:** ^1^ Department of Surgery Saga University Faculty of Medicine Japan; ^2^ Department of Surgery National Hospital Organization Higashisaga Hospital Saga Japan

**Keywords:** farnesyltransferase inhibitor, gastric cancer, HIF‐1α, mTOR, ROS, tipifarnib

## Abstract

Farnesyltransferase inhibitors (FTIs) suppress tumor aggressiveness in several malignancies by inhibiting Ras signaling. However, treatment of cells with a low dose of the FTI tipifarnib suppresses the expression of hypoxia‐inducible factor‐1α (HIF‐1α) and results in antitumor effects without inhibiting the Ras pathway. Although we previously reported that elevated HIF‐1α expression is associated with an aggressive phenotype in gastric cancer (GC), little is known about the antitumor effects of FTIs on GC. In this study, we examined the relationship between the antitumor effects of low‐dose tipifarnib and HIF‐1α expression in GC cells. Under normoxic conditions, HIF‐1α was expressed only in MKN45 and KATOIII cells. The inhibitory effect of tipifarnib on HIF‐1α was observed in HIF‐1α‐positive cells. Low‐dose tipifarnib had antitumor effects only on HIF‐1α‐positive cells both *in vitro* and *in vivo*. Furthermore, low‐dose tipifarnib inactivated ras homolog enriched in brain (Rheb)/mammalian target of rapamycin (mTOR) signaling and decreased intracellular reactive oxygen species (ROS) levels in HIF‐1α‐positive GC cells. Our results that the antitumor effects of low‐dose tipifarnib are at least partially mediated through suppression of mTOR signaling and HIF‐1α expression via inhibition of Rheb farnesylation and reduction in ROS levels. These findings suggest that low‐dose tipifarnib may be capable of exerting an antitumor effect that is dependent on HIF‐1α expression in GC cells. Tipifarnib may have potential as a novel therapeutic agent for HIF‐1α‐expressing GC exhibiting an aggressive phenotype.

## Graphical abstract

Low‐dose tipifarnib has antitumor effects on gastric cancer (GC) cells via suppression of mammalian target of rapamycin signaling and reactive oxygen species production. The effects of tipifarnib depend on hypoxia‐inducible factor‐1α (HIF‐1α) expression status. HIF‐1α expression is associated with cancer aggressiveness in GC. Tipifarnib may represent a novel therapeutic agent for GC exhibiting an aggressive phenotype.

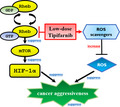

**Abbreviations**
4E‐BP: eukaryotic translation initiation factor 4E‐binding protein
ALDOC: fructose‐bisphosphate aldolase C
ANGPTL4: angiopoietin‐like 4
CA9: carbonic anhydrase 9
CAT: catalase
FTase: farnesyltransferase
FTI: farnesyltransferase inhibitor
GC: gastric cancer
GPX2: glutathione peroxidase 2
HIF‐1α: hypoxia‐inducible factor‐1α
Keap1: Kelch‐like ECH‐associated protein 1
LDHA: lactate dehydrogenase A subunit
mTOR: mammalian target of rapamycin
Nrf2: NF‐E2‐related factor 2
PDK1: dependent protein kinase 1
Rheb: ras homolog enriched in brain
ROS: reactive oxygen species
RT‐qPCR: quantitative real‐time PCR
SOD2: superoxide dismutase 2
VEGFA: vascular endothelial growth factor A

Gastric cancer (GC) is the sixth most common malignancy in the world and the second leading cause of cancer‐related deaths [1]. To date, the development of systemic chemotherapy and the incorporation of immunotherapy have improved the survival of patients with advanced GC. However, treatment of patients with advanced stage or potentially aggressive GC often results in poor survival because of metastasis and peritoneal dissemination [2, 3]. Therefore, a novel therapeutic strategy for GC exhibiting an aggressive phenotype is needed to improve clinical outcome.

Hypoxia‐inducible factor‐1α (HIF‐1α) is stabilized under hypoxic conditions and plays an essential role in oxygen homeostasis [4]. Previous studies have reported that HIF‐1α is stabilized by reactive oxygen species (ROS) generated within cells in a normoxic environment [5]. Furthermore, the translation efficiency of HIF‐1α is activated by the mammalian target of rapamycin (mTOR) signal pathway, intracellular calcium ion concentration, and protein kinase A (PKA) phosphorylation in an oxygen concentration‐independent manner [6]. HIF‐1α is a powerful transcriptional factor for cancer‐related genes, and it regulates energy metabolism, angiogenesis, apoptosis, and cell survival [7, 8, 9]. Therefore, the inhibition of HIF‐1α represents an attractive and promising strategy to improve cancer therapy [10, 11, 12]. We have determined the relationship of HIF‐1α with cancer aggressiveness and its potential role as a therapeutic target [13, 14, 15, 16, 17].

Protein farnesylation, a posttranslational protein prenylation modification, is catalyzed by farnesyltransferase (FTase) and is an essential step for the Ras maturation and function [18]. FTase inhibitors (FTIs) inhibit the Ras signaling pathway and the proliferation of many cancer and leukemia cell lines [19, 20]. Phase II and phase III clinical trials have been conducted to evaluate the safety and efficacy of FTIs in combination with other cytotoxic chemotherapeutic agents in cancer and hematologic malignancies [21, 22, 23]. However, there are no reports describing the effects of FTIs on GC cells *in vitro* or *in vivo*.

With respect to the effect of FTIs on HIF‐1α expression under normoxic conditions, it has been shown that a low dose (300 nm) of the FTI, tipifarnib decreases HIF‐1α expression, migration, and tumorsphere formation in human triple‐negative breast cancer cells (TNBCs) that are devoid of any hormone or human epidermal growth factor receptor 2 receptor under normoxic conditions *in vitro*. In addition, tipifarnib ameliorated the Warburg effect and suppressed the epithelial‐to‐mesenchymal transition [24]. Thus, FTIs may be an effective agent for TNBCs with elevated expression of HIF‐1α, which is associated with cancer aggressiveness. It has also been established that elevated HIF‐1α expression is associated with the aggressiveness of GC [13, 14, 15, 16, 17]. However, the antitumor effects of FTIs have not yet been studied in GC. These observations prompted us to study the effects of FTIs in MKN45 and KATOIII GC cells relative to HIF‐1α expression under normoxic conditions.

We determined whether clinically relevant, low‐dose tipifarnib could inhibit HIF‐1α expression and cancer aggressiveness. We also measured its effect on cell proliferation and migration in GC cell lines with HIF‐1α expression under normoxic conditions. In addition, we examined the mechanism of HIF‐1α inhibition by low‐dose tipifarnib with respect to ROS levels.

## Materials and methods

### Cell culture and reagents

To study the effects of FTIs on the HIF‐1α pathway, the GC cell line MKN45 (poorly differentiated adenocarcinoma), MKN74 (moderately differentiated type), and KATOIII (signet ring cell carcinoma) were selected for study. These cell lines were purchased from the RIKEN Cell Bank (Tsukuba, Japan). The cells were grown in complete culture medium [RPMI‐1640 medium (Sigma‐Aldrich, St. Louis, MO, USA) supplemented with 10% FBS (Biowest, Nuaillé, France) and 100 µg·mL^−1^ kanamycin (Meiji, Tokyo, Japan)] at 37 °C in a humidified atmosphere under normoxic conditions (5% CO_2_ in air). The cells were then treated with tipifarnib (R115777; Chemscene, Monmouth Junction, NJ, USA) at different concentrations for 24 h unless otherwise indicated. Stock solutions of tipifarnib (24 mm) were prepared using DMSO.

### Western blot analysis

Whole‐cell lysates were prepared by resuspending cells in lysis buffer [150 mm NaCl, 50 mm Tris/HCl, pH 7.5, 2 mm EDTA, 1% Triton X‐100, 1% sodium deoxycholate, 2% SDS, 28 µm PMSF, and a protease inhibitor cocktail mix (Roche, Mannheim, Germany)]. The lysates were sonicated for 30 s. Western blot analysis was performed as previously described [25]. In brief, aliquots containing 30 µg of protein were separated on 5–20% Bis/Tris gels (Intertechno, Tokyo, Japan) and transferred to Hybond ECL membranes (GE Healthcare, Little Chalfont, UK). Membranes were blocked with 5% skim milk or 2% BSA in Tris‐buffered saline containing 0.01% Tween 20 for 30 min and then incubated overnight at 4 °C with the following primary antibodies: anti‐HIF‐1α (1 : 1000, #610958; Becton Dickinson Biosciences, Franklin Lakes, NJ, USA), anti‐HDJ2 (1 : 1000, ab126774; Abcam, Cambridge, UK), anti‐ras homolog enriched in brain (Rheb; 1 : 1000, GTX54697; GeneTex, Irvine, CA, USA), anti‐phospho‐mTOR (1 : 1000, #5536; Cell Signaling Technology, Danvers, MA, USA), anti‐mTOR (1 : 1000, #2983; Cell Signaling Technology), anti‐phospho‐S6 ribosomal protein (1 : 1000, #5364; Cell Signaling Technology), anti‐S6 ribosomal protein (1 : 1000, #2217; Cell Signaling Technology), anti‐eukaryotic translation initiation factor 4E‐binding protein (4E‐BP; 1 : 1000, #9955; Cell Signaling Technology), anti‐phospho‐4E‐BP (1 : 1000, #2855; Cell Signaling Technology), anti‐NF‐E2‐related factor 2 (Nrf2; 1 : 500, sc‐365949; Santa Cruz Biotechnology, Dallas, TX, USA), anti‐Kelch‐like ECH‐associated protein 1 (Keap1; 1 : 1000, #8047; Cell Signaling Technology), and anti‐β‐actin (1 : 10 000, AC15; Sigma‐Aldrich). The signal was developed using ECL Prime Western Blotting Detection Reagent (GE Healthcare, Chicago, IL, USA). Images were acquired using a FUSION FX7.EDGE (Vilber‐Lourmat, Marne‐la‐Vallée, France).

### Cell proliferation assay

The effect of tipifarnib on cell proliferation was assessed by counting the number of viable cells using the trypan blue exclusion assay with a hemocytometer. Cells (1 × 10^5^) were seeded into 6‐cm culture dishes. After 18 h, they were treated with tipifarnib (300 nm or 3 μm) or vehicle alone (0.1% DMSO) for up to 72 h. Every 24 h, the culture media were replaced with fresh media and incubated with or without inhibitor.

### Wound‐healing assay

Migratory capacity was assessed by a wound‐healing assay using culture inserts (ibidi, Madison, WI, USA) in six‐well plates. After cells were cultured in the culture inserts overnight, the cells were treated with or without tipifarnib (300 nm) for 24 h. The culture inserts were then gently removed with sterile tweezers, and the cells were cultured with tipifarnib (300 nm) or vehicle alone (0.01% DMSO) for an additional 72–120 h. Wound closure was monitored by images captured with a microscope immediately after the removal of the culture inserts (0 h) and every 12 h thereafter. The rate of wound closure was quantified by ImageJ software.

### Animal experiments

The animal experimental protocols were approved by the Animal Care Committee of Saga University (Saga, Japan; Permission No. 30‐032‐0). Female athymic BALB/cAJcl mice (nu/nu, 4 weeks old) were obtained from Nihon Crea Co. (Tokyo, Japan). They were kept under specific pathogen‐free conditions and were provided with sterile food and water. The ambient light was controlled to provide regular 12‐h light and dark cycles. Approximately 5 × 10^6^ MKN74 and MKN45 cells were injected subcutaneously into both flanks of the mice. Thereafter, 15 mice (30 tumors) were evaluated per cell line. Once the tumor xenografts became palpable, the tumors were measured in two perpendicular dimensions with a caliper every day. The tumor size (*T*) was evaluated as the maximum cut area, determined by the following formula *T* = π/4 × *a* × *b*, where *a* was the shorter axis (mm) and *b* was the longer axis (mm). From day 7, the mice were treated with daily subcutaneous injections of 3 mg·kg^−1^·day^−1^ tipifarnib or vehicle (5% DMSO in 0.1 mL normal saline) alone for 2 or 4 weeks.

### Quantification of intracellular ROS levels by flow cytometry

Intracellular ROS levels were evaluated using a 2,7‐dichlorofluorescein diacetate‐based Total ROS Detection Kit (ENZO Life Sciences, Farmingdale, NY, USA), in accordance with the manufacturer's instructions. In brief, at the end of the experiment, cells (1 × 10^5^/sample) were washed and resuspended in ROS detection solution. Fluorescence was detected using a FACSCalibur flow cytometer (Becton Dickinson, San Jose, CA, USA) using excitation and emission wavelengths of 488 and 545 nm, respectively. Data were analyzed using FlowJo version 10.0 software (FlowJo, Ashland, OR, USA). All experiments were performed in triplicate. Data are displayed as the geometric mean fluorescence.

### Extraction of RNA and quantitative real‐time PCR analysis

Total RNA was extracted from cells using Isogen II (Nippon Gene, Osaka, Japan), and 1 μg aliquots of each RNA sample was reverse‐transcribed using a ReverTra Ace Kit (Toyobo, Osaka, Japan). Quantitative real‐time PCR (RT‐qPCR) was performed using a CFX Connect Real‐Time PCR Detection System (Bio‐Rad, Hercules, CA, USA) with SsoAdvanced Universal SYBR Green Supermix (Bio‐Rad), in accordance with the manufacturer's protocol. The PCR program was performed using a Two‐Step Amp procedure as described previously [25]. β‐actin mRNA served as an internal control. Primers for lactate dehydrogenase A subunit (LDHA), fructose‐bisphosphate aldolase C (ALDOC), carbonic anhydrase 9 (CA9), angiopoietin‐like 4 (ANGPTL4), dependent protein kinase 1 (PDK1), vascular endothelial growth factor A (VEGFA), superoxide dismutase 2 (SOD2), catalase (CAT), glutathione peroxidase 2 (GPX2), and β‐actin (ACTB) were as follows: LDHA forward, 5′‐GGC CTG TGC CAT CAG TAT CT‐3′, and reverse, 5′‐CTT TCT CCC TCT TGC TGA CG‐3′; ALDOC forward, 5′‐AAA TTG GGG TGG AAA ACA CA‐3′, and reverse, 5′‐AGA AAA TGA CGC CTC CAA TG‐3′; CA9 forward, 5′‐CCG AGC GAC GCA GCC TT TGA‐3′, and reverse, 5′‐GGC TCC AGT CTC GGC TAC CT‐3′; PDK1 forward, 5′‐GGT TAC GGG ACA GAT GCA GT‐3′, and reverse, 5′‐CGT GGT TGG TGT TGT AAT GC‐3′; VEGFA forward, 5′‐GGA GGG CAG AAT CAT CAC GA‐3′, and reverse; 5′‐TTG GTG AGG TTT GAT CCG CA‐3′; SOD2 forward, 5′‐TCT GTT GGT GTC CAA GGC TC‐3′, and reverse, 5′‐GCC TGT TGT TCC TTG CAG TG‐3′; CAT forward, 5′‐CTC CGG AAC AAC AGC CTT CT‐3′, and reverse, 5′‐ATA GAA TGC CCG CAC CTG AG‐3′; GPX2 forward, 5′‐TCC TGT CTT CGC CTAC CTG A‐3′, and reverse, 5′‐CCG GCC CTA TGA GGA ACT TC‐3′; and ACTB forward, 5′‐ACG CCT CTG GCC GTA CCA CT‐3′, and reverse, 5′‐TAA TGT CAC GCA CGA TTC CC‐3′. All experiments were performed in triplicate, and the mean values were calculated.

### Statistical analysis

All data are expressed as the means ± SEM. For comparisons between two groups, significant differences were evaluated by an unpaired, two‐tailed Student's *t*‐test. *P* < 0.05 was considered statistically significant. Data were analyzed using JMP Pro 14 software (SAS Institute, Inc., Cary, NC, USA).

### Ethics approval and consent to participate

An animal experimental protocol was reviewed and approved by the Animal Care Committee of Saga University (Saga, Japan; Permission No. 30‐032‐0).

## Results

### Tipifarnib decreases HIF‐1α expression in HIF‐1α‐positive GC cells under normoxia

**Fig. 1:**
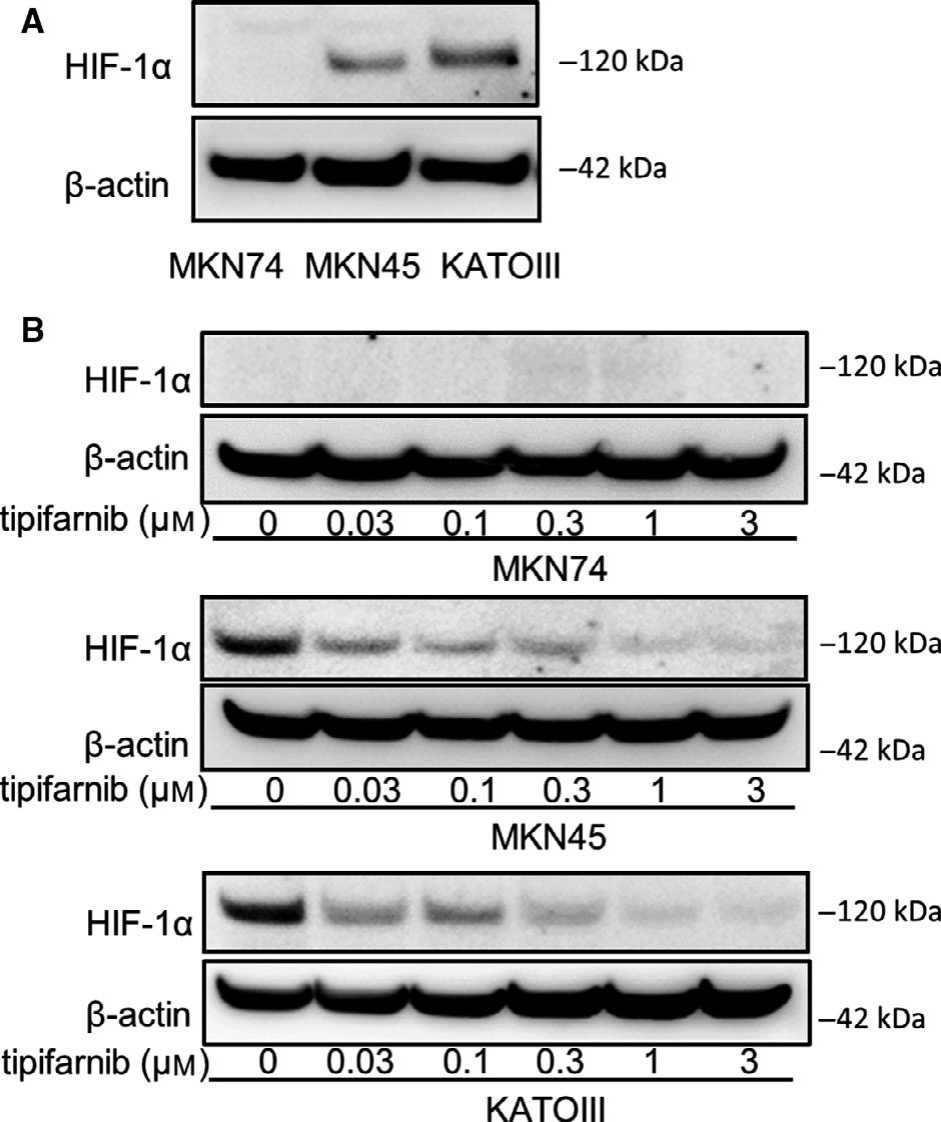
Effects of tipifarnib on HIF‐1α expression in GC cells. (A) HIF‐1α expression was observed in MKN45 and KATOIII under normoxic conditions, but not in MKN74 cells. (B) Treatment with tipifarnib for 24 h decreased HIF‐1α expression in a dose‐dependent manner.

**Fig. 2:**
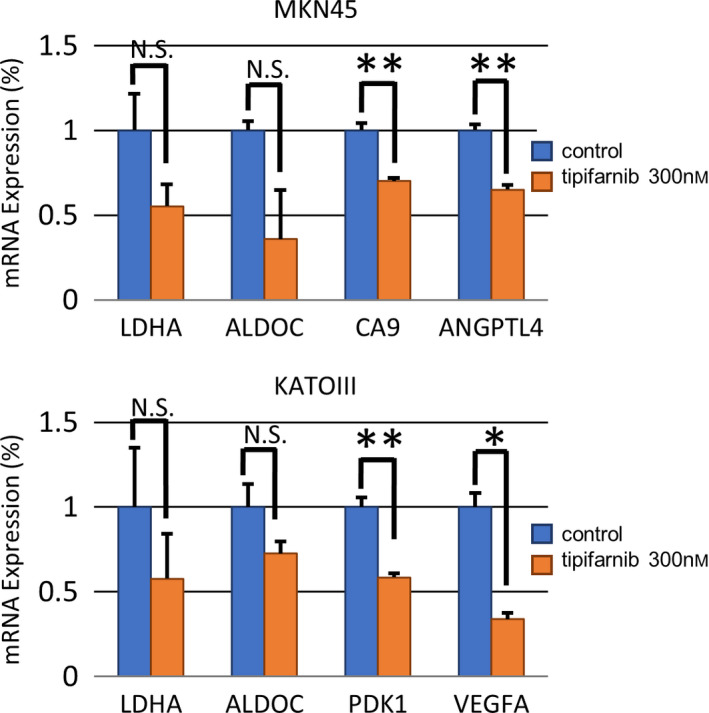
Effects of tipifarnib on mRNA expression of HIF‐1α's downstream genes. Effects of tipifarnib (300 nm) treatment for 24 h on mRNA levels of LDHA, ALDOC, CA9, PDK1, ANGPTL4, PDK1, and VEGFA downstream genes of HIF‐1α were evaluated by real‐time RT‐qPCR. Data are expressed as means ± SEM and are representative of three independent experiments and statistically analyzed by two‐tailed Student's *t*‐test. N.S.: not significant, *P < 0.05, **P < 0.01 versus control.

The expression of HIF‐1α was investigated in GC cell lines cultured under for 24 h under normoxic conditions. HIF‐1α expression was significantly elevated in MKN45 and KATOIII cells compared with MKN74 cells as determined by western blot analysis (Fig. 1A). Tipifarnib decreased HIF‐1α protein expression in a dose‐dependent manner in MKN45 and KATOIII cells. HIF‐1α expression in MKN45 and KATOIII cells was not detectable with tipifarnib at 3 μm. Tipifarnib did not affect HIF‐1α expression in MKN74 (Fig. 1B). Neither tipifarnib at 300 nm nor tipifarnib at 3 μm altered β‐actin protein expression in these cell lines. Furthermore, we showed the change in mRNA levels of HIF‐1α target genes such as LDHA, PDK1, ALDOC, VEGFA, and ANGPTL4 in MKN45 and KATOIII by RT‐qPCR (Fig. 2). These genes were key factors in tumor proliferation, migration, and cancer energy metabolism. The mRNA levels of these genes were decreased in MKN45 and KATOIII by the treatment of tipifarnib at 300 nm for 24 h.

### Tipifarnib decreased cell proliferation in HIF‐1α‐positive GC cells

**Fig. 3:**
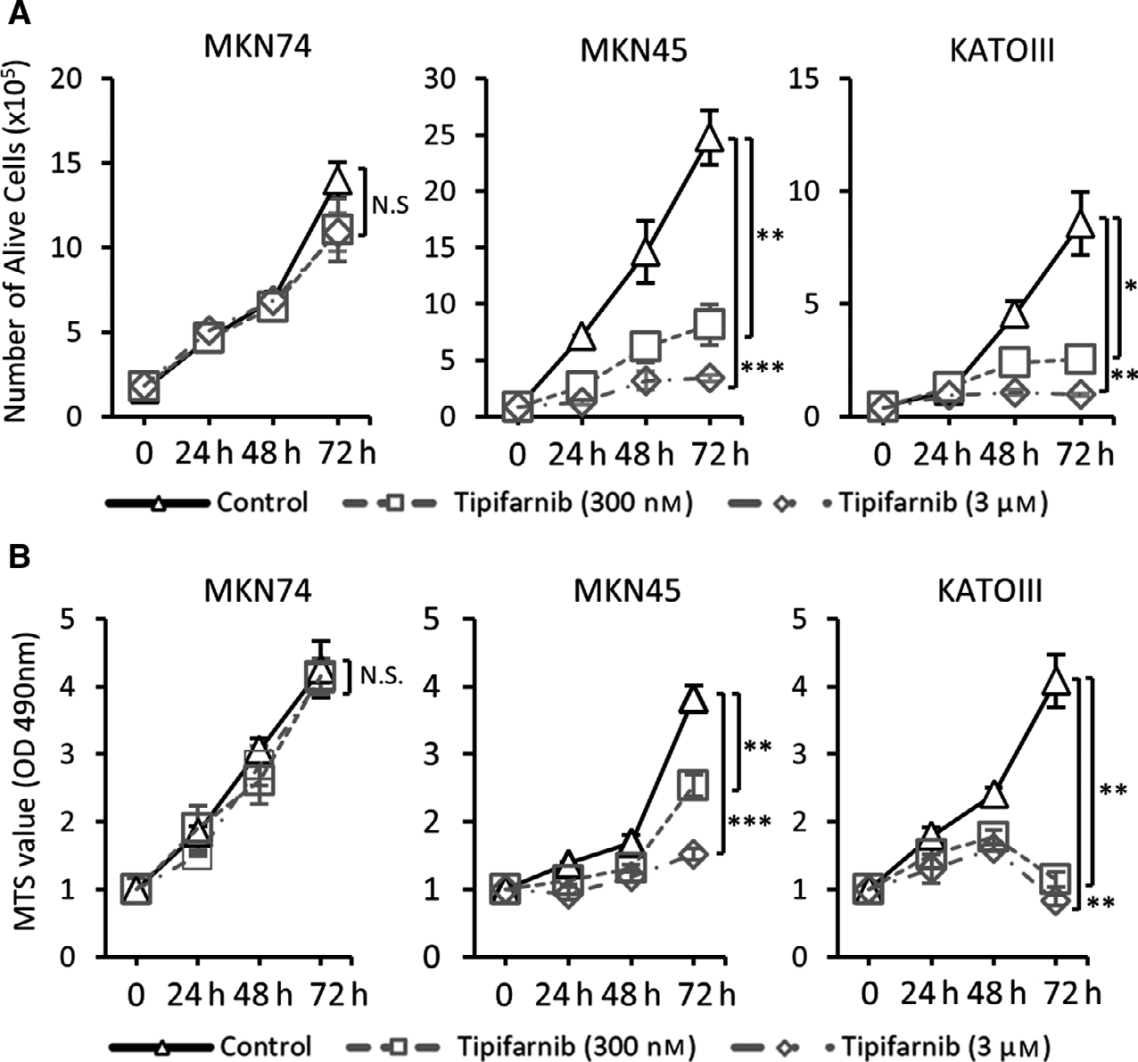
Effects of tipifarnib on cell proliferation in GC cells. Trypan blue exclusion assay (A) and MTS assay (B) revealed that tipifarnib inhibited cell growth in a dose‐dependent manner only in MKN45 and KATOIII cells. Values are presented as the mean ± SEM of three independent experiments and statistically analyzed by two‐tailed Student's *t*‐test. N.S.: not significant, *P < 0.05, **P < 0.01, ***P < 0.001 versus control.

We examined the effects of tipifarnib on cell proliferation in three GC cells under normoxic conditions. Trypan blue and MTS assays showed that treatment with tipifarnib at both 300 nm and 3 μm inhibited cell growth for up to 72 h in MKN45 and KATOIII cells. However, tipifarnib had no effect on cell proliferation in MKN74 at any dose (Fig. 3). These results suggested that tipifarnib decreased cell proliferation only in HIF‐1α‐positive GC cells.

### Tipifarnib inhibits the migration capabilities of HIF‐1α‐positive GC cells

**Fig. 4:**
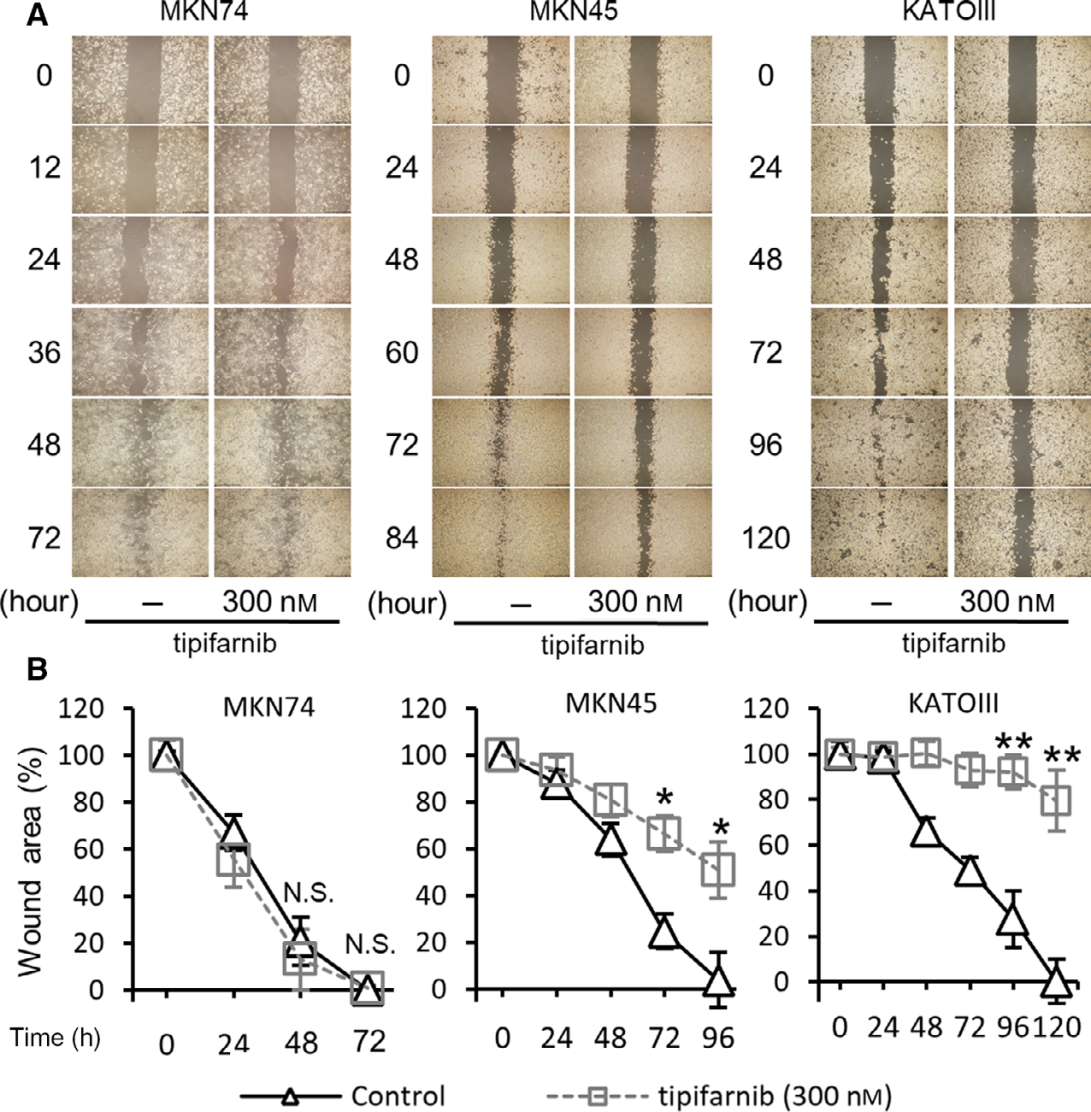
Tipifarnib inhibited migration of GC cells. A wound‐healing assay revealed that tipifarnib (300 nm) significantly inhibited migration in MKN45 and KATOIII cells, as judged by a reduction in wound area. Scale bars, 400 µm. Data are expressed as means ± SEM and are representative of three independent experiments and statistically analyzed by two‐tailed Student's *t*‐test. N.S.: not significant, *P < 0.05, **P < 0.01 versus control.

A wound‐healing assay revealed that tipifarnib inhibited migration of MKN45 and KATOIII cells significantly for up to 96 h or 120 h (Fig. 4). However, tipifarnib did not affect migration of MKN74 cells. These results indicate that tipifarnib inhibits the migration of HIF‐1α‐positive GC cells.

### Tipifarnib suppresses the growth of HIF‐1α‐positive GC cell tumor xenografts in nude mice

**Fig. 5:**
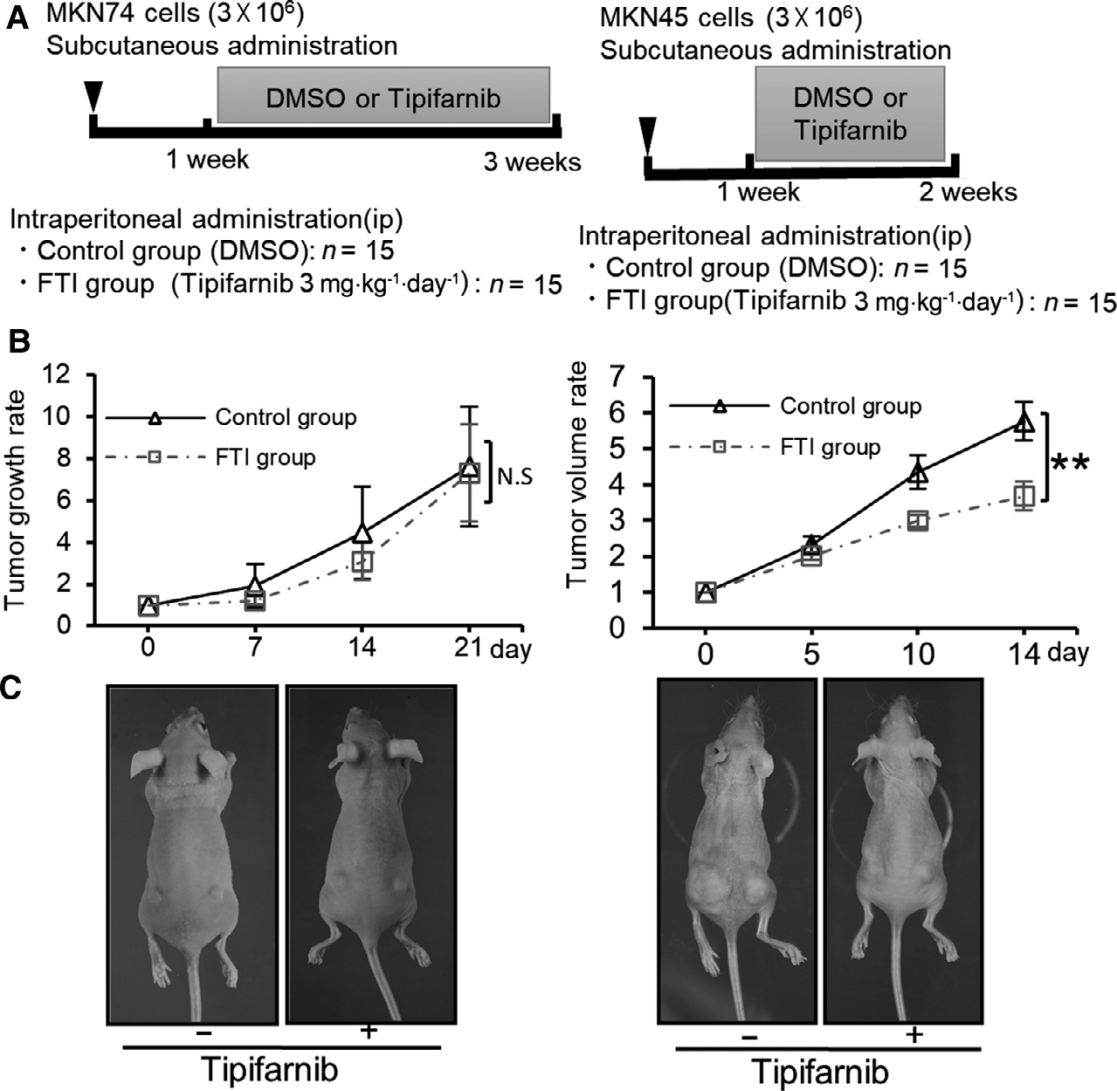
The *in vivo* effect of tipifarnib treatment on tumor xenografts. (A) The experimental schedule of MKN74 or MKN45 (*n* = 15/group) tumors treated with DMSO or tipifarnib. (B) Representative images of MKN74 or MKN45 tumors treated with DMSO or tipifarnib. (C) Tumor sizes in the control and tipifarnib groups were estimated on the indicated days. The mean value ± SEM of tumor volumes was calculated and plotted on the graph and statistically analyzed by two‐tailed Student's *t*‐test. N.S.: not significant, **P < 0.01 versus control.

We determined the effect of tipifarnib on GC tumor xenografts *in vivo*. MKN74, MKN45, and KATOIII cells were injected subcutaneously into the backs of nude mice. As KATOIII cells (signet ring cell carcinoma) did not form any subcutaneous nodule, the animal experiments were limited to MKN74 and MKN45 cells. Figure 5A demonstrates the experimental design of the xenograft murine model after subcutaneous injection of MKN74 or MKN45 cells, in the backs of nude mice. Subsequently, vehicle or tipifarnib (3 mg·kg^−1^) was injected intraperitoneally into the mice bearing GC tumors. Representative images of the tumor‐bearing mice that were treated with vehicle or tipifarnib are shown in Fig. 5B. The sizes of the MKN74 tumors were similar between the control and tipifarnib groups. Conversely, the MKN45 cell tumors in the tipifarnib group were significantly smaller than those in the control group (Fig. 5C).

### Tipifarnib inhibits activation of the mTOR pathway in GC cell lines

**Fig. 6:**
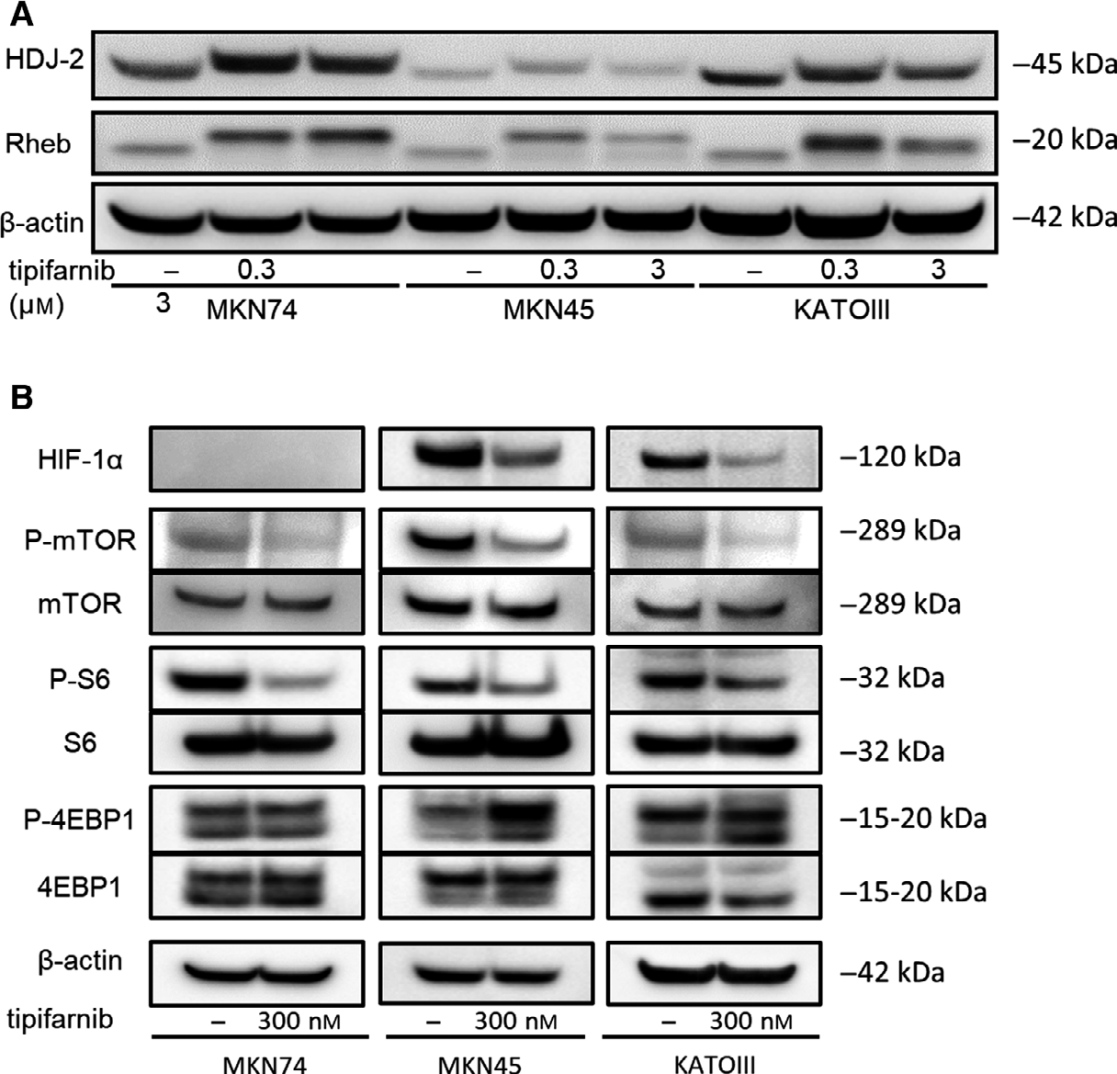
Effects of tipifarnib on farnesylation inhibition in GC cells. (A) Tipifarnib treatment for 24 h inhibited farnesylation of HDJ2 and Rheb in MKN74, MKN45, and KATOIII cells. (B) Effects of low‐dose tipifarnib on the mTOR signaling pathway in GC cells. Tipifarnib (300 nm) treatment for 24 h altered the phosphorylation and protein expression of mTOR, S6k1, and 4E‐BP1 in MKN74, MKN45, and KATOIII cells.

To further investigate the inhibitory effect of tipifarnib on GC cells, we assessed farnesylation of the HDJ2 and Rheb proteins. Figure 6A shows that the farnesylation activity in MKN74, MKN45, and KATOIII cells was inhibited by 300 nm and 3 μm tipifarnib. To investigate the molecular events underlying the inhibition of HIF‐1α expression and the antitumor effects of FTIs, we measured mTOR signaling downstream of Rheb following tipifarnib treatment. Tipifarnib inhibited mTOR phosphorylation in the three GC cell lines. Activation of the mTOR/S6k and mTOR/4E‐BP1 pathways was inhibited by tipifarnib. Therefore, the expression of HIF‐1α in MKN45 and KATOIII cells was inhibited (Fig. 6B).

### Tipifarnib had different effects on the antioxidant response in GC cells

**Fig. 7:**
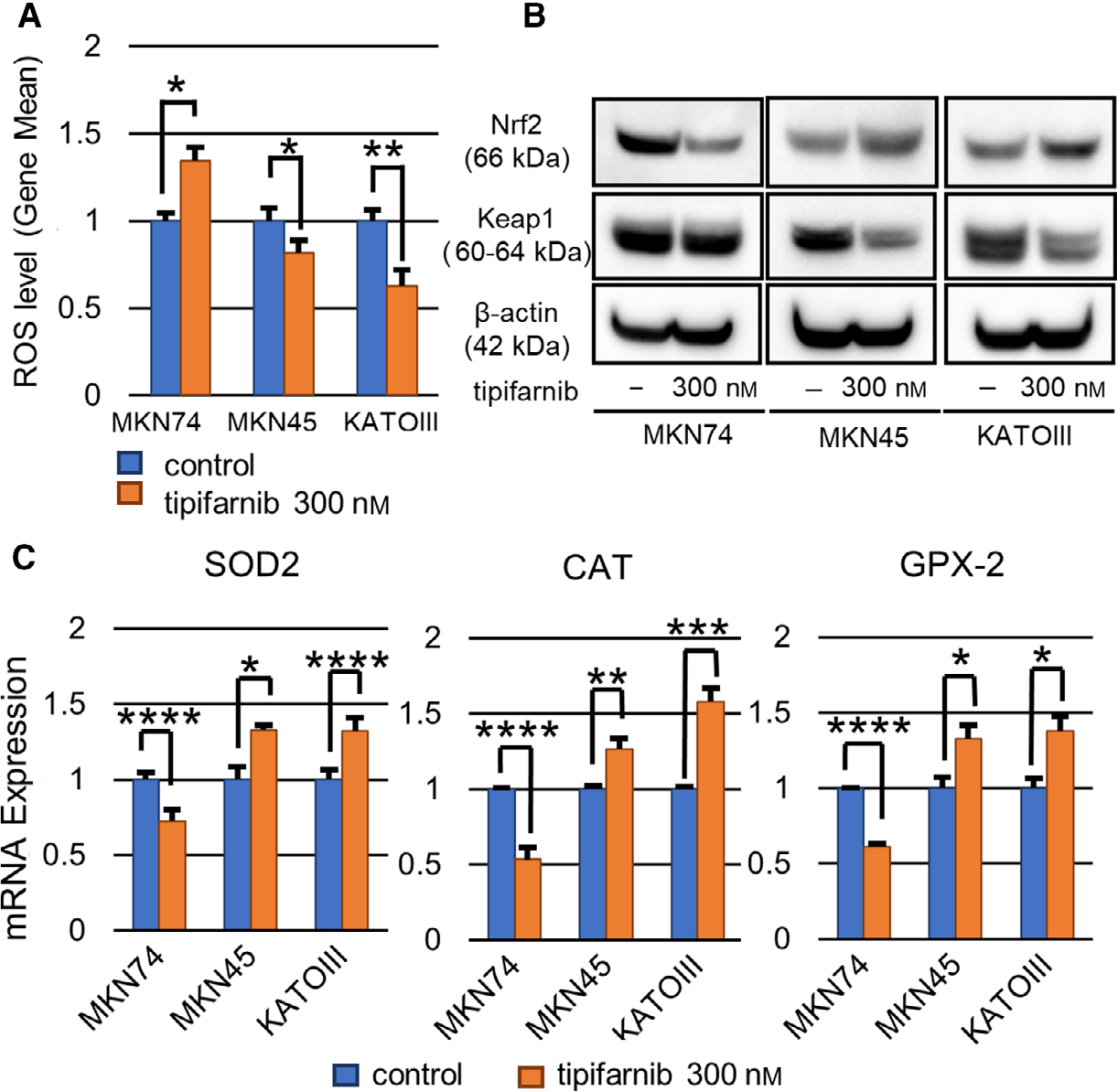
Tipifarnib suppressed the antioxidant response in MKN74, but promoted it in MKN45 and KATOIII cells. (A) Tipifarnib treatment for 48 h decreased ROS levels in MKN45 and KATOIII cells. (B) Tipifarnib activated the Keap1‐Nrf2 pathway and increased antioxidant enzyme expression. (C) Conversely, tipifarnib decreased antioxidant enzyme expression and increased ROS levels in MKN74 cells. Data are expressed as means ± SEM and are representative of three independent experiments and statistically analyzed by two‐tailed Student's *t*‐test. *P < 0.05, **P < 0.01, ***P < 0.001, ****P < 0.0001 versus control.

To investigate the difference in response to tipifarnib in GC cells, we evaluated the effects of tipifarnib on ROS levels. Tipifarnib reduced ROS levels in MKN45 and KATOIII cells, whereas tipifarnib increased ROS in MKN74 cells (Fig. 7A). In addition, the Nrf2/Keap1 pathway, which regulates antioxidant enzymes, was activated in MKN45 and KATOIII cells (Fig. 7B). The expression of SOD2, CAT, and GPX2, which are representative antioxidant enzymes, was induced by tipifarnib (Fig. 7C).

## Discussion

In this study, low‐dose (300 nm) tipifarnib exerted antitumor effects on HIF‐1α‐positive GC cell lines both *in vitro* and *in vivo*. In contrast, it did not show any effect on a HIF‐1α‐negative GC cell line. These findings strongly suggest that the antitumor effect of low‐dose tipifarnib may be dependent on HIF‐1α expression. Therefore, inhibition of FTase by low‐dose tipifarnib exhibits an antitumor effect by decreasing HIF‐1α expression in MKN45 and KATOIII cells, which are HIF‐1α‐positive GC cell lines. Low‐dose tipifarnib inhibits Rheb farnesylation, which suppresses the mTOR/HIF‐1α pathway. Furthermore, low‐dose tipifarnib reduced ROS levels through the induction of antioxidant enzymes and subsequent activation of the Nrf2/Keap1 pathway in HIF‐1α‐positive GC cells.

In this study, we mainly performed *in vitro* experiment under normoxic condition. The reason is that solid cancers also have heterogeneity for oxygen partial pressure, as well as the gene expression and blood distribution. Of note, previous studies reported that the induction or stabilization of HIF‐1α occurs by increased ROS, mTOR signal pathway, the concentration of intracellular calcium ion, and the phosphorylation by protein kinase A in an oxygen concentration‐independent manner [5, 6]. Moreover, it was also reported that cancers with high malignant potential such as triple‐negative breast cancer show HIF‐1α expression even under normoxic environment [24]. Furthermore, a low‐dose tipifarnib suppressed the cell proliferation in MKN74 cells under the hypoxic condition. A hypoxic environment induced HIF‐1α expression in even if MKN 74 which is low‐HIF‐1α expressing GC cells under normoxia, however, a low‐dose tipifarnib suppressed HIF‐1α expression in MKN74 under hypoxic condition (data not shown). These results would also support that the antitumor effect of FTI, tipifarnib, depends on HIF‐1α expression.

In previous studies, high‐dose tipifarnib (> 5 μm) exerted an antitumor effect by inhibiting RAS signaling [26, 27]. Another study showed that 300 nm tipifarnib decreased HIF‐1α expression, migration, and tumorsphere formation in human triple‐negative breast cancer cells without inhibiting the RAS pathway [24]. Thus, the antitumor effects of low‐dose tipifarnib might be dependent on HIF‐1α expression. The antitumor effects observed at high tipifarnib doses (> 5 μm) may be the result of inhibition of RAS signaling irrespective of the presence or absence of HIF‐1α expression. It is well known that HIF1‐α expression is strongly associated with an aggressive phenotype of cancer that includes metastasis and chemoresistance [13, 15]. Therefore, low‐dose tipifarnib is expected to be effective only for cancers with a highly malignant phenotype. The results of pharmacokinetic studies in phase I clinical trials of tipifarnib (200 or 300 mg, b.i.d.) in advanced cancer patients indicated that the average circulating concentrations of the drug reached approximately 500 nm to 1.5 mm [28, 29, 30]. Compared with these studies, the dose of tipifarnib used in our study was clinically relevant both *in vitro* and *in vivo*.

The mTOR protein is associated with various pathways that regulate glucose or oxygen levels, and it plays a central role in the regulation of nutrition and energy signals [31]. This serine/threonine kinase binds to multiple proteins to form two distinct complexes, mTOR complexes 1 and 2 (mTORC1 and mTORC2). Each complex recognizes various molecules and performs different biological functions [32, 33]. For example, mTORC1 activates the synthesis of proteins, including HIF‐1α, by phosphorylating p70S6 kinase (S6K) and 4E‐BP [34, 35]. It also suppresses autophagy, a process that degrades unnecessary cytoplasmic components and organelles, and lysosomal function [36, 37, 38]. Activation of mTORC1 requires GTP bound to Rheb (Rheb‐GTP) [38]. Of note, the activation of Rheb requires farnesylation. Thus, FTI inhibits mTORC1 activation by inactivating Rheb. In addition, several studies reported that Rheb was activated by farnesylation independently of the RAS pathway. Furthermore, FTIs did not inhibit RAS activity, but suppressed the mTOR pathway by inhibiting the farnesylation of Rheb [27, 39]. In this study, we demonstrated that low‐dose tipifarnib inhibited the farnesylation of Rheb and simultaneously suppressed phosphorylation of mTOR and the downstream target genes of mTORC1, namely S6K and 4E‐BP1. Our data suggest that the suppression of mTOR/HIF‐1α through the inhibition of Rheb farnesylation is a component of the antitumor mechanism of low‐dose tipifarnib.

Next, we addressed the mechanism through which tipifarnib exerts its antitumor effects in a HIF‐1α‐dependent manner. To clarify the underlying mechanism, we focused on cancer energy metabolism, in particular, ROS. Several reports have demonstrated that ROS stabilize HIF‐1α and contribute to cancer aggressiveness in an oxygen concentration‐independent manner [40, 41, 42]. It is well known that ROS are regulated by various factors. Under hypoxic conditions, ROS production in mitochondria increases. It is also reported that ROS are hydrolyzed by antioxidant enzymes induced by autophagy [43, 44]. In our study, tipifarnib reduced ROS levels in the HIF‐1α‐positive GC cells, MKN45 and KATOIII, whereas it increased ROS in the HIF‐1α‐negative MKN74 cell line. We hypothesize that the altered ROS levels in GC cells after treatment with tipifarnib were related to its antitumor mechanism of the action. Exposure of MKN45 and KATOIII cells to low‐dose tipifarnib, which suppressed ROS levels, inhibited cell proliferation and migration. In contrast, there was no significant tipifarnib antitumor effect in MKN74 cells, which did not exhibit decreased ROS levels. Therefore, ROS levels are a key factor in the antitumor effects of low‐dose tipifarnib and decreased ROS levels lead to the suppression of HIF‐1α expression and cancer aggression.

Moreover, we showed that the effect of reduced ROS levels in MKN45 and KATOIII cells by tipifarnib was associated with Nrf2/Keap1 pathway activation, which is required for the induction of antioxidant enzymes. In contrast, antioxidant enzyme expression was decreased after treatment with tipifarnib in MKN74 cells. Thus, it is possible that the status of HIF‐1α expression may be related to an increase or decrease in antioxidant enzymes following exposure to low‐dose tipifarnib.

In conclusion, this study suggests that FTase represents a novel target for GC therapy. Low‐dose tipifarnib is a promising treatment to control GC with an aggressive phenotype that exhibits high HIF‐1α expression by regulating Rheb farnesylation, mTOR/HIF‐1α signaling, and ROS levels.

## Author contributions

NE, TT, and YK conceived and designed the experiments. NE performed the majority of the experiments. SM, KY, KI, HK, and KO performed certain experiments. NE and TT analyzed the data. NE and TT contributed to the interpretation of the data and were major contributors in writing the manuscript. HN contributed to the research design and edited the manuscript. All authors read and approved the final manuscript.

## Conflict of interest

The authors declare no conflict of interest.

## Data Availability

The data sets generated during the study are available from the corresponding author on reasonable request.

## References

[1] Bray F , Ferlay J , Soerjomataram I , Siegel RL , Torre LA and Jemal A (2018) Global cancer statistics 2018: GLOBOCAN estimates of incidence and mortality worldwide for 36 cancers in 185 countries. CA Cancer J Clin 68, 394–424. 30207593 10.3322/caac.21492

[2] Shiraishi N , Inomata M , Osawa N , Yasuda K , Adachi Y and Kitano S (2000) Early and late recurrence after gastrectomy for gastric carcinoma. Univariate and multivariate analyses. Cancer 89, 255–261. 10918153 10.1002/1097-0142(20000715)89:2<255::aid-cncr8>3.0.co;2-n

[3] Macdonald JS , Smalley SR , Benedetti J , Hundahl SA , Estes NC , Stemmermann GN , Haller DG , Ajani JA , Gunderson LL , Jessup JM *et al*. (2001) Chemoradiotherapy after surgery compared with surgery alone for adenocarcinoma of the stomach or gastro‐esophageal junction. N Engl J Med 345, 725–730. 11547741 10.1056/NEJMoa010187

[4] Wang GL and Semenza GL (1993) General involvement of hypoxia‐inducible factor 1 in transcriptional response to hypoxia. Proc Natl Acad Sci USA 90, 4304–4308. 8387214 10.1073/pnas.90.9.4304 PMC46495

[5] Movafagh S , Crook S and Vo K (2015) Regulation of hypoxia‐inducible Factor‐1a by reactive oxygen species: new developments in an old debate. J Cell Biochem 116, 696–703. 25546605 10.1002/jcb.25074

[6] Bullen JW , Tchernyshyov I , Holewinski RJ , DeVine L , Wu F , Venkatraman V , Kass DL , Cole RN , Van Eyk J and Semenza GL (2016) Protein kinase A‐dependent phosphorylation stimulates the transcriptional activity of hypoxia‐inducible factor 1. Sci Signal 9, ra56. 27245613 10.1126/scisignal.aaf0583 PMC5541497

[7] Semenza GL (2002) HIF‐1 and tumor progression: pathophysiology and therapeutics. Trends Mol Med 8, 62–67. 11927290 10.1016/s1471-4914(02)02317-1

[8] Kitajima Y and Miyazaki K (2013) The critical impact of HIF‐1α on gastric cancer biology. Cancers 5, 15–26. 24216696 10.3390/cancers5010015 PMC3730315

[9] Wang F , Chang M , Shi Y , Jiang L , Zhao J , Hai L , Sharen G and Du H (2014) Down‐regulation of hypoxia‐inducible factor‐1 suppresses malignant biological behavior of triple‐negative breast cancer cells. Int J Clin Exp Med 7, 3933–3940. 25550901 PMC4276159

[10] Burroughs SK , Kaluz S , Wang D , Wang K , Van Meir EG and Wang B (2013) Hypoxia inducible factor pathway inhibitors as anticancer therapeutics. Future Med Chem 5, 553–572. 23573973 10.4155/fmc.13.17 PMC3871878

[11] Georgina NM and Wei L (2015) HIF‐1α pathway: role, regulation and intervention for cancer therapy. Acta Pharm Sin B 5, 378–389. 26579469 10.1016/j.apsb.2015.05.007 PMC4629436

[12] Nable DG and Zhou YD (2006) Natural product‐based inhibitors of hypoxia‐inducible factor‐1 (HIF‐1). Curr Drug Targets 7, 355–369. 16515532 10.2174/138945006776054979 PMC2908043

[13] Nakamura J , Kitajima Y , Kai K , Hashiguchi K , Hiraki M , Noshiro H and Miyazaki K (2010) HIF‐1α is an unfavorable determinant of relapse in gastric cancer patients who underwent curative surgery followed by adjuvant 5‐FU chemotherapy. Int J Cancer 127, 1158–1171. 20020496 10.1002/ijc.25129

[14] Hiraki M , Kitajima Y , Kai K , Nakamura J , Hashiguchi K , Noshiro H and Miyazaki K (2012) Knockdown of hypoxia‐inducible factor‐1 α accelerates peritoneal dissemination via the upregulation of MMP‐1 expression in gastric cancer cell lines. Exp Ther Med 4, 355–362. 23181099 10.3892/etm.2012.600 PMC3503539

[15] Miyake S , Kitajima Y , Nakamura J , Kai K , Yanagihara K , Tanaka T , Hiraki M , Miyazaki K and Noshiro H (2013) HIF‐1α is a crucial factor in the development of peritoneal dissemination via natural metastatic routes in scirrhous gastric cancer. Int J Oncol 43, 1431–1440. 23970191 10.3892/ijo.2013.2068

[16] Tanaka T , Kitajima Y , Miyake S , Yanagihara K , Hara H , Nishijima‐Matsunobu A , Baba K , Shida M , Wakiyama K , Nakamura J *et al*. (2015) The apoptotic effect of HIF‐1α inhibition combined with glucose plus insulin treatment on gastric cancer under hypoxic conditions. PLoS One 10, e0137257. 26339797 10.1371/journal.pone.0137257 PMC4560388

[17] Baba K , Kitajima Y , Miyake S , Nakamura J , Wakiyama K , Sato H , Okuyama K , Kitagawa H , Tanaka T , Hiraki M *et al*. (2017) Hypoxia‐induced ANGPTL4 sustains tumour growth and anoikis resistance through different mechanisms in scirrhous gastric cancer cell lines. Sci Rep 7, 11127. 28894280 10.1038/s41598-017-11769-x PMC5594024

[18] Zhang FL and Casey PJ (1996) Protein prenylation: molecular mechanisms and functional consequences. Annu Rev Biochem 65, 241–269. 8811180 10.1146/annurev.bi.65.070196.001325

[19] Smalley KSM and Eisen TG (2002) Farnesyl thiosalicylic acid inhibits the growth of melanoma cells through a combination of cytostatic and pro‐apoptotic effects. Int J Cancer 98, 514–522. 11920610 10.1002/ijc.10213

[20] Shen M , Pan P , Li Y , Li D , Yu H and Hou T (2015) Farnesyltransferase and geranylgeranyltransferase I: structures, mechanism, inhibitors and molecular modeling. Drug Discov Today 20, 267–276. 25450772 10.1016/j.drudis.2014.10.002

[21] Jabbour E , Kantarjian H , Ravandi F , Garcia‐Manero G , Estrov Z , Verstovsek S , O’Brien S , Faderl S , Thomas DA , Wright JJ and *et al*. (2011) A phase 1–2 study of a farnesyltransferase inhibitor, tipifarnib, combined with idarubicin and cytarabine for patients with newly diagnosed acute myeloid leukemia and high‐risk myelodysplastic syndrome. Cancer 117, 1236–1244. 20960519 10.1002/cncr.25575 PMC4061136

[22] Li T , Guo M , Gradishar WJ , Sparano JA , Perez EA , Wang M and Sledge GW (2012) A phase II trial of capecitabine in combination with the farnesyltransferase inhibitor tipifarnib in patients with anthracycline‐treated and taxane‐resistant metastatic breast cancer: an Eastern Cooperative Oncology Group Study (E1103). Breast Cancer Res Treat 134, 345–352. 22547107 10.1007/s10549-012-2071-z PMC4596817

[23] Andreopoulou E , Vigoda IS , Valero V , Hershman DL , Raptis G , Vahdat LT , Han HS , Wright JJ , Pellegrino CM , Cristofanilli M *et al*. (2013) Phase I‐II study of the farnesyl transferase inhibitor tipifarnib plus sequential weekly paclitaxel and doxorubicin‐cyclophosphamide in HER2/neu‐negative inflammatory carcinoma and non‐inflammatory estrogen receptor‐positive breast carcinoma. Breast Cancer Res Treat 141, 429–435. 24068539 10.1007/s10549-013-2704-x PMC3999640

[24] Tanaka T , Ikegami Y , Nakazawa H , Kuriyama N , Oki M , Hanai J , Sukhatme VP and Kaneki M (2017) Low‐dose farnesyltransferase inhibitor suppresses HIF‐1α and snail expression in triple‐negative breast cancer MDA‐MB‐231 cells *in vitro* . J Cell Physiol 232, 192–201. 27137755 10.1002/jcp.25411

[25] Sato H , Hiraki M , Namba T , Egawa N , Baba K , Tanaka T and Noshiro H (2018) Andrographolide induces degradation of mutant p53 via activation of Hsp70. Int J Oncol 53, 761–770. 29845212 10.3892/ijo.2018.4416

[26] Nam JS , Ino Y , Sakamoto M and Hirohashi S (2002) Ras farnesylation inhibitor FTI‐277 restores the E‐cadherin/catenin cell adhesion system in human cancer cells and reduces cancer metastasis. Jpn J Cancer Res 93, 1020–1028. 12359056 10.1111/j.1349-7006.2002.tb02479.x PMC5927130

[27] Basso AD , Mirza A , Liu G , Long BJ , Bishop WR and Kirschmeier P (2005) The farnesyl transferase inhibitor (FTI) SCH66336 (lonafarnib) inhibits Rheb farnesylation and mTOR signaling: Role in FTI enhancement of taxane and tamoxifen anti‐tumor activity. J Biol Chem 280, 31101–31108. 16006564 10.1074/jbc.M503763200

[28] Cohen SJ , Ho L , Ranganathan S , Abbruzzese JL , Alpaugh RK , Beard M , Lewis NL , McLaughlin S , Rogatko A , Perez‐Ruixo JJ *et al*. (2003) Phase II and pharmacodynamic study of the farnesyltransferase inhibitor R115777 as initial therapy in patients with metastatic pancreatic adenocarcinoma. J Clin Oncol 21, 1301–1306. 12663718 10.1200/JCO.2003.08.040

[29] Johnston SR , Hickish T , Ellis P , Houston S , Kelland L , Dowsett M , Salter J , Michiels B , Perez‐ Ruixo JJ , Palmer P *et al*. (2003) Phase II study of the efficacy and tolerability of two dosing regimens of the farnesyl transferase inhibitor, R115777, in advanced breast cancer. J Clin Oncol 21, 2492–2499. 12829668 10.1200/JCO.2003.10.064

[30] Lebowitz PF , Eng‐Wong J , Widemann BC , Balis FM , Jayaprakash N , Chow C , Clark G , Gantz SB , Venzon D and Zujewski J (2005) A phase I trial and pharmacokinetic study of tipifarnib, a farnesyltransferase inhibitor, and tamoxifen in metastatic breast cancer. Clin Cancer Res 11, 1247–1252. 15709195

[31] Hirao A and Hoshii T (2013) Mechanistic/mammalian target protein of rapamycin signaling in hematopoietic stem cells and leukemia. Cancer Sci 104, 977–982. 23648144 10.1111/cas.12189 PMC7657242

[32] Ma XM and Blenis J (2009) Molecular mechanisms of mTOR‐mediated translational control. Rev Mol Cell Biol 10, 307–318. 10.1038/nrm2672 19339977

[33] Meric‐Bernstam F and Gonzalez‐Angulo AM (2009) Targeting the mTOR signaling network for cancer therapy. J Clin Oncol 27, 2278–2287. 19332717 10.1200/JCO.2008.20.0766 PMC2738634

[34] Jiang BH , Jiang G , Zheng JZ , Lu Z , Hunter T and Vogt PK (2001) Phosphatidylinositol 3‐kinase signaling controls levels of hypoxia‐inducible factor 1. Cell Growth Differ 12, 363–369. 11457733

[35] Semenza G (2002) Signal transduction to hypoxia‐inducible factor 1. Biochem Pharmacol 64, 993–998. 12213597 10.1016/s0006-2952(02)01168-1

[36] Albert V and Hall MN (2015) mTOR signaling in cellular and organismal energetics. Curr Opin Cell Biol 33, 55–66. 25554914 10.1016/j.ceb.2014.12.001

[37] Huang K and Fingar DC (2014) Growing knowledge of the mTOR signaling network. Semin Cell Dev Biol 36, 79–90. 25242279 10.1016/j.semcdb.2014.09.011 PMC4253687

[38] Takahara T and Maeda T (2013) Evolutionarily conserved regulation of TOR signaling. J Biochem 154, 1–10. 23698095 10.1093/jb/mvt047

[39] Niessner H , Beck D , Sinnberg T , Lasithiotakis K , Maczey E , Gogel J , Venturelli S , Berger A , Mauthe M , Toulany M *et al*. (2011) The farnesyl transferase inhibitor lonafarnib inhibits mTOR signaling and enforces sorafenib‐induced apoptosis in melanoma cells. J Invest Dermatol 131, 468–479. 20944654 10.1038/jid.2010.297

[40] Chandel NS , McClintock DS , Feliciano CE , Wood TM , Melendez JA , Rodriguez AM and Schumacker PT (2000) Reactive oxygen species generated at mitochondrial complex III stabilize hypoxia‐inducible factor‐1alpha during hypoxia: a mechanism of O2 sensing. J Biol Chem 275, 25130–25138. 10833514 10.1074/jbc.M001914200

[41] Brunelle JK , Bell EL , Quesada NM , Vercauteren K , Tiranti V , Zeviani M , Scarpulla RC and Chandel NS (2005) Oxygen sensing requires mitochondrial ROS but not oxidative phosphorylation. Cell Metab 1, 409–414. 16054090 10.1016/j.cmet.2005.05.002

[42] Koshikawa N , Hayashi J , Nakagawara A and Takenaga K (2009) Reactive oxygen species‐generating mitochondrial DNA mutation up‐regulates hypoxia‐inducible factor‐1alpha gene transcription via phosphatidylinositol 3‐kinase‐Akt/protein kinase C/histone deacetylase pathway. J Biol Chem 284, 33185–33194. 19801684 10.1074/jbc.M109.054221 PMC2785161

[43] Klimova T and Chandel NS (2008) Mitochondrial complex III regulates hypoxic activation of HIF. Cell Death Differ 15, 660–666. 18219320 10.1038/sj.cdd.4402307

[44] Reuter S , Gupta SC , Chaturvedi MM and Aggarwal BB (2010) Oxidative stress, inflammation, and cancer: How are they linked? Free Radic Biol Med 49, 1603–1616. 20840865 10.1016/j.freeradbiomed.2010.09.006 PMC2990475

